# Mobility patterns and associated factors among pregnant internal migrant women in China: a cross-sectional study from a National Monitoring Survey

**DOI:** 10.1186/s12884-018-1813-2

**Published:** 2018-05-15

**Authors:** Ying Ji, Xiaoping Zhao, Zhili Wang, Shenglan Liu, Yang Shen, Chun Chang

**Affiliations:** 1 Department of Social Medicine and Health Education, School of Public Health, Peking University, Beijing, China; 2 Service Center for Immigrant of National Health and Family Planning Commission of China, Beijing, China; 3 China Population and Development Research Center, Beijing, China

**Keywords:** Mobility during pregnancy, Internal migrant, Maternal health, Social integration, Delivery location

## Abstract

**Background:**

Residential instability during pregnancy has been linked to poor health outcomes. As a first step toward providing better health care to pregnant migrant women, the size and characteristics of this population and factors associated with mobility during pregnancy should be studied.

**Methods:**

Using the “Monitoring Data of Chinese Migrants” for 2012, from the Chinese National Population and Family Planning Commission, this study explored mobility patterns during pregnancy and associated factors among migrants within China. From a library of 158,556 participants, two subsamples were selected. Percentages, with chi-squared tests, and means and standard deviations, with ANOVAs, were adopted to describe mobility patterns during pregnancy (*always staying in sending area, mainly staying in sending area, mainly staying in receiving area,* and *always staying in receiving area*) and delivery location choice. Logistic regression analysis was used to explore the associated factors.

**Results:**

We found that the percentage of migrants always or mainly staying in receiving areas during pregnancy rose from nearly 40% in 1985 to more than 80% in 2012, while the percentage of migrants who were mobile between receiving and sending areas during pregnancy fluctuated between 30 and 40% before 1995, and between 40 and 45% after 1995, decreasing to around 40% after 2008. The percentage of respondents who chose to deliver in receiving areas fluctuated but increased from 10% in 1985 to more than 50% in 2011. Among respondents who had delivered during the last year of the survey period, families with older pregnant women (OR = 1.09, 95% CI 1.05–1.13), their own housing (OR = 5.66, 95% CI 2.45–13.05), longer time in the receiving area (OR = 1.14, 95% CI 1.09–1.20), and strong will to integrate (OR = 1.32, 95% CI 1.15–1.51) always stayed in the receiving area during pregnancy, rather than the sending area, and families with broadly similar characteristics were inclined to choose the receiving area for their delivery.

**Conclusions:**

The mobility patterns of pregnant migrant women in China have been changing in recent years, with the percentage of them staying in receiving areas during pregnancy and delivering there increasing. Individual and family characteristics were also associated with mobility patterns and delivery location choice.

**Electronic supplementary material:**

The online version of this article (10.1186/s12884-018-1813-2) contains supplementary material, which is available to authorized users.

## Background

Pregnancy is a critical period for the health of both mothers and infants, and mobility during pregnancy raises public health concerns given that residential instability has been linked to poor health outcomes of children and mothers. First, mobility during pregnancy brings environmental change that may be associated with congenital anomalies and children with asthma [1–5]. Second, mobility during pregnancy may lead to decreased use of maternal health care services due to less knowledge and information, financial barriers, or other barriers to accessibility of services [6–11], although it is important from the outcomes perspective to utilize and maintain regular contact with health services during pregnancy to reduce the risk of low birth weight, preterm births, and perinatal deaths [6, 12–15]. Third, mobility during pregnancy is detrimental to health care provision. Failure to address the scale and traits of these populations and their specific demands impedes appropriate service provision [7, 16–20]. In addition to these factors, in China, inadequate health information tracing and discontinuity of pregnancy health records contribute much to the difficulties of service provision. Given the deficiency of referral mechanisms in China, pregnant women might choose any accessible hospital for their delivery, which might lead to considerable unexpected pressure on large hospitals. Therefore, describing the trends of and factors affecting mobility during pregnancy is necessary.

Internal migrants are a special population in China. “Migrant” is an identity applied to those leaving their residence for a certain period without changing their household registration, or *hukou*. The *hukou* system in China has segregated the rural and urban populations in geographical terms. Established in the late 1950s, it was initially a primary means of controlling population mobility and determining eligibility for public services and welfare, such as access to local public schools, pension plans, and public housing. *Hukou* conversion, meaning change from the rural to the urban category or from a small city to a large one, was controlled and permitted only under limited conditions [21]. After the mid-1980s, however, large-scale rural-to-urban migration became possible. A relaxation in the implementation of *hukou* laws and the re-commodification of many basic goods meant that the rapidly growing urban private sector was able to absorb large numbers of laborers from small cities and the countryside. An unprecedented wave of large-scale migration had begun. Although the mobility of people is no longer tightly regulated and migrants without local *hukou* in receiving areas can access to more public welfare than before, transfer of *hukou* status is still subject to strict policy and quota controls, especially in large cities [22]. Although the *hukou* system is specific to China, internal migrants have other characteristics in common with migrants in China and other developing countries, such as low education, rural origins, and heavy work stress [18, 22–25]. Among migrants in developing countries, the utilization of antenatal care services in receiving areas is inadequate [18, 26, 27]. The reasons for low utilization rates of maternal health care by migrants are various, including poor health knowledge and awareness, financial or cultural barriers, and provider factors [7, 16, 18–20, 23]. This population migration has brought increased attention to the issue of migrant health, as illustrated for example by the World Health Organization’s calling upon its member states to promote migrant-sensitive health policies, equal access to health promotion, and disease prevention, especially for migrant women and children [28].

As mentioned above, both mobility behavior during pregnancy and the identity characteristics of migrants can influence their health care utilization and provisions. In our study, we define *migrant* as an identity applied to those leaving their residence for a certain period without changing their household registration, and *mobility* as a migrant’s travel behavior between sending and receiving areas. During pregnancy and delivery, migrants may either go back to their original registration area (sending area), stay in the receiving area, or move to a new area (new receiving area). Therefore, as a first step to providing better health care to pregnant migrant women, policymakers should familiarize themselves with the scale, characteristics, and change trends of the target population and the factors associated with their movements. Many studies from developed countries have explored mobility during pregnancy in the general population [1, 2, 29, 30], and family and individual characteristics have been analyzed to detect associated factors [1, 8, 29]. However, few studies have explored mobility during pregnancy among internally migrant population in developing countries.

In this context, the purpose of this study was to analyze mobility patterns in China’s internal migrant population during pregnancy, including factors associated with it.

## Methods

### Data source

Cross-sectional data were obtained from the 2012 “Monitoring Data of Chinese Migrants” survey conducted in China and published by the National Population and Family Planning Commission (containing data for 2011). Inclusion criteria for the survey were males and females living in their local area for 1 month or longer without local household registration and aged between 15 and 59 years as of May 2012. Exclusion criteria were those having a spouse or child from a local household (those whose spouse or child holds local household registration are not deemed migrants, as their families can access local public welfare services, such as entering public school and applying for public housing) or those who were part of the temporary floating population at railway stations, docks, airports, hotels, and hospitals. Only one person in each household was surveyed. The survey contained items for respondents’ demographic characteristics and those of their family members, employment, housing, health care, marital status, obstetrical status, and level of social integration. This adopted stratified sampling for migrants from each province and a three-stage sampling method with probability proportional to unit size within the province (town/subdistrict, village/community, and individual). The samples were representative nationally as well as for each province [31]. (See Additional file 1).

The sample size of the survey was 158,556 people. The survey method used was face-to-face interviews, with community staff filling out questionnaires and importing data through an electronic system. The study has been granted an exemption from the Peking University Institutional Review Board (No. IRB00001052–16011).

### Selection of samples for analysis

From the library of 158,556 individual samples, 29,720 cases (18.74%) who were married, had borne children, and had had migration experience before the conception of their first child were chosen. Within the shortlisted cases, 185 cases who had had children between 1970 and 1984 were excluded due to their relatively small number, as their inclusion may have led to data instability. Furthermore, 771 cases who had given birth between January and May of 2012 and thus did not have a full year of data available were excluded, leaving 28,764 cases from which to analyze the mobility patterns of migrants during the gestational and peripartum period of their first child. Thus, from the library of 158,556 samples, 4176 cases (2.63%) who were married and had borne children between June 2011 and May 2012, the last year of the survey period, were chosen; they were mothers who had had migration experience before conception, and thus were a fitting sample from which to analyze the mobility characteristics of migrants during pregnancy. The respondents selected were women aged 17–49.

### Measures

Based on previous studies, we hypothesized that the socioeconomic status (SES) of the family, family migration characteristics, parity (that is, first or not first child), and social integration situation were associated with mobility during pregnancy.

Family SES includes the *education level of the pregnant woman*, the family *Engel coefficient* (hereafter EC, the percentage of household expenditure devoted to food), *whether they have their own house in the receiving area*, and *whether family members have urban employee medical insurance*. The EC was considered to be a measure of effective wealth; since the income gaps between provinces in China are big and the levels of consumption are different, it is difficult to compare absolute value of income. In our study, two questions were used to calculate EC: “How much does your family spend monthly (in RMB) on local food?” and “What is your monthly household income (in RMB)?” The minimum EC value was 0.01 and the maximum value was 1.00, with a mean of 0.55 (medium value 0.56) and a standard deviation of 0.19. In other studies, EC has been used as an indirect reflection of SES [32, 33]; as demand for food is inelastic, poorer families have a higher EC.

*Owning a house in the receiving area* was considered as a covariate. In the late 1990s, with development of the market economy in China, owning a house ceased to be strictly limited by the household registration system—that is, people could buy a house without local household registration. Since 2010, however, to control the rapidly increasing price of houses, some local governments in big cities have imposed restrictions on buying houses for people without a local household registration, even though migrants might have their own houses owing to the different housing policies among cities. For example, in Beijing migrants are eligible to buy houses if they have been working in Beijing for more than 5 years, provided they submit certification of continuous income taxes. Some types of houses are unrestricted, such as houses used for both commerce and living (*ShangzhuLiangyong*). Given this background, owning a house reflects the following choices: whether a migrant family bought a house early (before 2010), whether they worked in the city for a long time, and/or whether they became rich enough to buy a permitted house.

Family migration characteristics include *rural household registration, range of migration* (cross-province, cross-city within province, cross-district within city), and *migration time* (time since the person migrated into the local area). The *hukou* system in China has segregated not only the rural and urban populations but also various geographical populations at levels as small as subdistrict (urban)/town (rural). In our study, we used “*rural household registration*” (yes or no) to demonstrate rural–urban or urban–urban migration status (for our survey carried out in cities, no urban–rural or rural–rural migration was included) and adopted *migration range* (cross-province, cross-city within province, cross-district within city) as another variable demonstrating migration status.

*Status of social integration* (*SSI*) was determined by eight questions (see Table 1). Factor analysis was adopted, and two factors were extracted to explain the 50.3% variance, with *KMO* = 0.816 and *p* < 0.001 for Bartlett’s test. “Participation in local activities” and “With whom do you have the most contact?” were the two items that contributed more to Factor 1; we named these factors together the *level of participation*. The other six items demonstrated the feelings of migrants in the receiving areas and contributed more to Factor 2. We viewed this factor as a good proxy for will to integrate and named it accordingly as *will to integrate*. The Cronbach’s alpha of the overall SSI factor was 0.71, with 0.54 for *level of participation* and 0.75 for *will to integrate*. In the factor analysis, the means and standard deviations of the original variables were standardized to 0 and 1, respectively; higher scores indicated better integration status.

**Table 1:** Eight questions to measure status of social integration

	Items	Assignment methods
1	Have you or your family participated in cultural and sports activities, social activities, family planning association activities, health education activities, and elections in the local community this year?	A score from 0 to 5 based on participation in these five items
2	During your spare time, with whom do you have the most contact, locally?	Rarely have contact with people = 0, fellow villagers whose household registration is still in sending areas = 1, fellow villagers who are registered permanent residents in the local area = 2, and locals = 3; range from 0 to 3
3	I like the city I am living in presently	The answers “completely disagree,” “disagree,” “somewhat agree,” and “completely agree,” respectively assigned values 1, 2, 3, and 4
4	I pay attention to the changes in the city I am living in	
5	I am willing to blend in with the locals	
6	I think locals are willing to accept me	
7	I feel that locals despise outsiders	The answers “completely disagree,” “disagree,” “somewhat agree,” and “completely agree,” respectively assigned values 4, 3, 2, and 1
8	Compared to the sending area, do you feel happy now?	The answers “very happy,” “happy,” “so-so,” “unhappy,” and “very unhappy,” respectively assigned values 5, 4, 3, 2, and 1

Mobility during pregnancy was assessed by investigating mobility patterns and selection of delivery location. Mobility patterns were grouped into *always staying in the sending area* (AS), *mainly staying in the sending area* (MS), *mainly staying in the receiving area* (MR), and *always staying in the receiving area* (AR). Delivery locations included *receiving area* (RA) and *sending area* (SA).

### Data analysis

Categorical variables were described using percentages and underwent a chi-squared test. Continuous variables were described using means and standard deviations and underwent an analysis of variance (ANOVA) with thresholds of *a* = 0.05 and *p* < 0.05. Logistic regression analysis demonstrated the influence of factors associated with mobility patterns and delivery location choice. Analyses were carried out using SPSS 13.0 (SPSS Inc., Chicago, IL).

## Results

### Retrospective analysis of 28,764 respondents’ mobility during pregnancy with their first child and of their delivery location

**Fig. 1:**
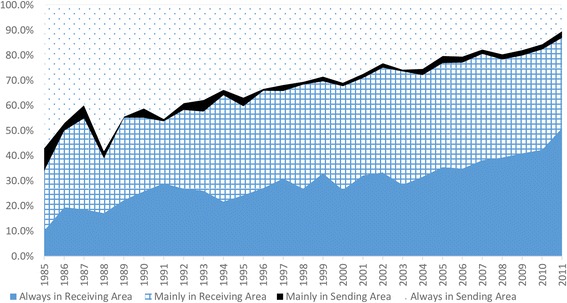
Mobility patterns during pregnancy among migrants in China (1985–2011)

As Fig. 1 indicates, for respondents expecting their first child, the rate of AS decreased and the rate of AR increased, while MS continually occupied a very low percentage, mostly less than 5%, and the rate of MR fluctuated while gradually increasing, only to decrease slightly after 2010. Meanwhile, it was found that the percentage of respondents who stayed in the receiving area (including AR and MR) increased from nearly 40% in 1985 to more than 80% in 2012. The percentage of migrants who were mobile during pregnancy (including MR and MS) fluctuated between 30 and 40% before 1995 and between 40 and 45% after 1995, decreasing to just under 40% after 2008.

**Fig. 2:**
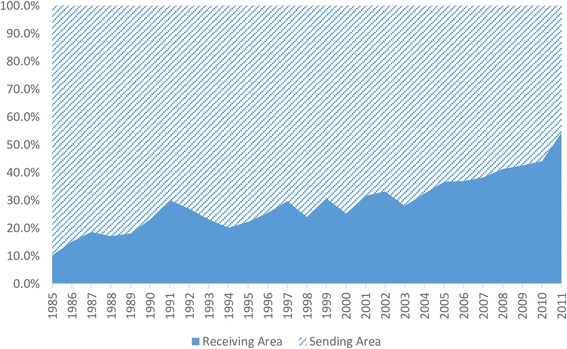
Choices of delivery location among migrants in China (1985–2011)

As Fig. 2 indicates, the percentage of respondents choosing to deliver in receiving areas increased each year; in 1985, only 10% of pregnant women delivered in a receiving area, but by 2011, this number had reached more than 50%.

### Respondents’ mobility patterns during pregnancy and choice of delivery location, among 4176 respondents who bore children between June 2011 and may 2012

Table 2 indicates that among these 4176 respondents, two-thirds had an education level below junior high school and an average EC of more than 50%. Only a few people had their own housing and/or urban medical insurance. The vast majority were from rural sending areas with rural household registration, and more than half of the children were the first child. Cross-provincial migration was the type of migration engaged in by 70.4% of respondents, and the average migration time was 3.77 years. Families with older pregnant women (compared with younger), their own housing (compared with not), from urban *sending areas* (compared with rural ones), whose children were not the first child (compared with the first child), and who had a cross-district *migration range* (compared with cross-province and cross-city), a longer *migration time* (compared with shorter), and/or a higher level of *participation* (compared with lower) had a higher rate of AR and a higher rate of delivery in the receiving area. Conversely, people with no *housing of their own* in the receiving area (compared with housing of their own), from rural *sending areas* (compared with urban areas), in their first *parity* (compared with not the first child), whose *migration range* was cross-city within the province or cross-province (compared with a merely cross-district migration range), having a moderate *migration time* (compared with shorter or longer), and with a moderate level of *participation* (compared with lower or higher) had a higher rate of mobility during pregnancy (including MR and MS).

**Table 2:** Characteristics of respondents; comparison of migrant mobility patterns during pregnancy and delivery locations (*n* (%), *m* ± SD)

		Mobility patterns					Delivery locations			Total
		AS	MS	MR	AR	*p*	RA	SA	*p*	*N* = 4176
		*N* = 305	*N* = 80	*N* = 1329	*N* = 2461		*N* = 2585	*N* = 1591		
Age (years)		25.90 ± 4.10	26.82 ± 4.23	27.16 ± 4.39	27.90 ± 4.88	< 0.001	27.89 ± 4.82	26.87 ± 4.43	< 0.001	27.50 ± 4.70
Education Level	Junior high school and below	214(7.7)	59(2.1)	878(31.4)	1641(58.8)	0.22	1719(61.6)	1073(38.4)	0.45	2793(67.0)
	Senior high school and above	87(6.3)	21(1.5)	447(32.6)	817(59.5)		862(62.8)	511(37.2)		
Engel Coefficient		0.54 ± 0.19	0.60 ± 0.21	0.54 ± 0.19	0.55 ± 0.19	0.03	0.55 ± 0.19	0.54 ± 0.19	0.24	0.55 ± 0.19
Own housing in RA	Yes	7(1.5)	7(1.5)	70 (14.8)	388(82.2)	< 0.001	403(85.0)	71(15.0)	< 0.001	473(11.3)
	No	297(8.0)	73(2.0)	1258(34.0)	2073(56.0)		2182(58.9)	1521(41.1)		
Urban employee Insurance	Yes	66(6.9)	22(2.3)	296(31.1)	569(59.7)	0.66	597(62.6)	357(37.4)	0.62	954(22.8)
	No	239(7.4)	52(1.8)	1032(32.0)	1892(58.7)		1988(61.7)	1234(38.3)		
Rural household registration	Yes	251(7.5)	64(1.9)	1099(33.0)	1916(57.5)	0.002	2006(60.2)	1325(39.8)	< 0.001	3331(80.1)
	No	50(6.1)	16(1.9)	225(27.2)	535(64.8)		569(68.9)	257(31.1)		
First parity	Yes	202(8.1)	52(2.1)	815(32.7)	1424(57.1)	0.017	1500(60.2)	992(39.8)	0.012	2493(59.7)
	No	103(6.1)	28(1.7)	513(30.5)	1037(61.7)		1084(64.4)	599(35.6)		
Range of migration	Cross-province	214(7.4)	54(1.9)	890(30.9)	1720(59.8)	< 0.001	1819(63.2)	1059(36.8)	< 0.001	2878(70.4)
	Within province cross-city	60(6.6)	13(1.4)	343(37.8)	491(54.1)		508(56.0)	399(44.0)		907(22.2)
	Within city cross-district	16(5.2)	7(2.3)	67(21.9)	216(70.6)		227(74.4)	78(25.6)		
Migration time	(years)	2.33 ± 3.00	2.94 ± 3.40	3.39 ± 3.54	4.17 ± 3.94	< 0.001	0.08 ± 1.02	−0.12 ± 0.96	< 0.001	3.77 ± 3.79
Level of participation		−0.09 ± 0.91	−0.06 ± 0.94	−0.05 ± 0.97	0.04 ± 1.03	0.03	4.14 ± 3.91	3.16 ± 3.50	< 0.001	0.00 ± 1.00
Will to integrate		− 0.31 ± 0.96	0.23 ± 1.03	− 0.05 ± 0.97	0.06 ± 1.01	< 0.001	0.03 ± 1.02	−0.05 ± 0.96	0.02	0.00 ± 1.00

*AS* always staying in the sending area, *MS* mainly staying in the sending area, *MR* mainly staying in the receiving area, *AR* always staying in the receiving area, *RA* receiving area, *SA* sending area

As Table 3 indicates, *will to integrate* was the only consistently significant factor associated with staying in the receiving area during pregnancy, mobility during pregnancy, and choosing to deliver in the receiving area. Other factors were only significant in some models. Families with older *age of pregnant women* (OR = 1.09, 95% CI 1.05–1.13), having their *own housing* (OR = 5.66, 95% CI 2.45–13.05), with a longer *migration time* (OR = 1.14, 95% CI 1.09–1.20), and with a higher level of *will to integrate* (OR = 1.32, 95% CI 1.15–1.51) were more likely to stay in the receiving area rather than the sending area during pregnancy. Among the associated factors, having one’s own housing had the greatest impact. Families with older *age of pregnant women* (OR = 1.06, 95% CI 1.02–1.10), a longer *migration time* (OR = 1.10, 95% CI 1.05–1.16), and a higher level of *will to integrate* (OR = 1.26, 95% CI 1.10–1.45) were more likely to spend most of their time during pregnancy in the receiving area or to be mobile between the sending area and receiving area, instead of staying in the sending area all or most the time, and *will to integrate* had stronger impact on them than on other families. Similarly, families with their *own housing*, a smaller *range of migration*, a longer *migration time*, and a higher level of *will to integrate* more often chose to deliver in the receiving area instead of the sending area; however, having their own housing was still the factor that had the most impact.

**Table 3:** Logistic regression analysis of mobility patterns during pregnancy and delivery location choice

		Mobility pattern (AS = 0)						AR = 0		Delivery locations	
		MS		MR		AR		MR		(SA = 0)	
		OR	95% C.I.	OR	95% C.I.	OR	95% C.I.	OR	95% C.I.	OR	95% C.I.
Age (years)	continuous	1.05	0.98, 1.12	1.06**	1.02, 1.10	1.09**	1.05, 1.13	0.98**	0.96, 0.99	1.04**	1.02, 1.06
Educational years	continuous	0.94	0.72, 1.23	1.08	0.93, 1.24	1.01	0.88, 1.15	1.07	0.99, 1.15	0.96	0.90, 1.03
Engel Coefficient	continuous	8.31**	1.97, 35.01	1.23	0.62, 2.45	1.45	0.74, 2.83	0.84	0.58, 1.22	1.12	0.79, 1.59
Own housing in RA	NO = 0	2.96	0.91, 9.69	1.81	0.76, 4.29	5.66**	2.45, 13.05	0.32**	0.24, 0.43	3.47**	2.60, 4.64
Urban Employees Insurance	NO = 0	1.23	0.65, 2.33	0.84	0.60, 1.19	0.71*	0.55, 0.99	1.18	0.43, 1.98	0.79*	0.66, 0.94
Rural household registration	NO = 0	1.04	0.47, 2.28	1.29	0.86, 1.94	1.06	0.71, 1.56	1.21	0.97, 1.50	0.83	0.68, 1.03
The first parity	NO = 0	1.25	0.65, 2.40	1.03	0.74, 1.42	1.02	0.75, 1.40	1.00	0.85, 1.19	0.99	0.85, 1.17
Range of migration	Cross-province	0.99	0.36, 2.69	1.19	0.66, 2.13	0.83	0.48, 1.44	1.42*	1.05, 1.91	0.73*	0.55, 0.97
Cross-district = 0	Cross-city	0.70	0.23, 2.15	1.54	0.82, 2.89	0.70	0.39, 1.28	2.17**	1.05, 1.91	0.46**	0.34, 0.62
Migration time	continuous	1.05	0.96, 1.15	1.10**	1.05, 1.16	1.14**	1.09, 1.20	0.96**	0.94, 0.98	1.05**	1.03, 1.07
Level of participation	continuous	0.98	0.75, 1.28	1.01	0.88, 1.16	1.08	0.94, 1.24	0.93	0.87, 1.00	1.06	0.99, 1.14
Will to integrate	continuous	1.89**	1.43, 2.49	1.26**	1.10, 1.45	1.32**	1.15, 1.51	0.95	0.89, 1.03	1.14**	1.06, 1.21

*AS* always staying in the sending area, *MS* mainly staying in the sending area, *MR* mainly staying in the receiving area, *AR* always staying in the receiving area, *RA* receiving area, *SA* sending area

*p < 0.05, **p < 0.01

Patterns of mobility during pregnancy and choice of delivery location were not statistically related to the *education level* of the pregnant woman, *rural residency*, *parity*, or level of *participation*.

## Discussion

This study found that of 28,764 respondents who had their first child between 1985 and 2011, the percentage always or mainly staying in receiving areas during pregnancy (AR and MR) increased over the years, while the percentage mobility during pregnancy (MS and MR) first increased and then decreased. The percentage of respondents who chose to deliver in receiving areas increased, reaching more than 50% in 2011.

Among the 4176 respondents who gave birth in the last year of the survey, families with older *age of pregnant women*, longer *length of stay in the receiving area*, and a stronger *will to integrate* were more likely to be AR or MR than others, but not AS. Similarly, families with older *age of pregnant women*, their *own housing*, small *migration range*, longer *length of stay in the receiving area*, and a stronger *will to integrate* chose the receiving area instead of the sending area as their delivery place. *Owning housing* in RA remained the factor with the most impact throughout.

Studies from other countries have indicated that 10–30% of pregnant women from the general population will be mobile during pregnancy [1, 2, 11]. The present study focused on people who had already had migration experience in China and demonstrated that the percentage of pregnant women who were mobile has been between 30 and 45% over the past 30 years, higher than among the general population in most other countries. However, because different countries’ backgrounds vary, the data are not appropriate for comparison. We speculate that in China, once women have migration experience, they prefer to be mobile during pregnancy in order to receive better family care or save on costs. Furthermore, we have little data from the Chinese general population, and our conclusion that the percentage of pregnant migrant women who are mobile is higher than that of pregnant women in general needs to be confirmed. Our results might surprise clinicians who had experience with a much higher mobility rate during pregnancy among migrants, and an environmental risks evaluation and prenatal examination reminders might be provided to the target population to provide better care.

Our results showed that migrant mobility patterns during pregnancy have been shifting from going back to sending areas once pregnant to staying in receiving areas for a longer time, even for the whole duration of the pregnancy. The rate of mobility during pregnancy (including MR and MS) began to fall in 2008, and the percentage of respondents choosing to deliver in receiving areas increased. Health care demands (such as antenatal and delivery care) decreased in sending areas but increased in receiving areas, since there were more and more pregnant migrants staying in receiving areas. As demonstrated in the last column in Table 2, rural residents accounted for 80.1%, and cross-provincial migration reached 70.4% of all respondents. Further analysis showed that 80.7% of cross-provincial migrants moved to eastern areas from central and western areas. In general, health resources quality and quantity are better in eastern China than in central and western areas (as is as economic level), as most health resources were allocated referring to the population distribution, especially to the household registration population, rather than all residents (household registration population and migrants) in China. More and more migrants inclining to give birth in receiving areas might bring challenges for health care provision, especially for overloaded hospitals in big cities. For example, according to 2012 health statistics, daily visits per doctor in China were 9.0, 5.6, and 6.7 for eastern, central, and western areas, respectively [34]. Gathering more information on the size and change trends of the population of pregnant migrant women is essential for policymakers and health care providers to make health care plans.

It has been shown that women who are young or who have fewer children, a lower level of education, lower household income, and do not own their own housing are more likely to be mobile during pregnancy [1, 8, 29]. The logistic regression analysis conducted in this study also confirmed that in internal migrant populations, young women without their own housing in RA are more likely to be mobile, similar to the findings of previous studies. The results should be used by clinicians to take note of pregnant women with these characteristics because of their higher possibility of mobility. In our results, though the mean differences in age were statistically significant, the value was between 25.90 and 27.90, thus with only limited clinical implications. In our analysis, contrary to the previous studies, it was also found that the education level of pregnant women and whether they were having their first child were not statistically significant. Furthermore, based on EC results, poor families are more likely to be mobile instead of staying in the sending area during pregnancy. Urban employee medical insurance is one of the three types of medical insurance the Chinese government provides for employees; having this insurance indicates that the family is of a high SES. This study showed that people with this insurance in RA were more likely to stay in their hometown or deliver in their hometown; this result did not match our expectation and needs further analysis.

In migrant populations, SSI’s impact on health and health behaviors has been demonstrated [35–37]. However, the definitions of SSI used by these studies have not been uniform. Different studies have evaluated social integration in terms of language, economic characteristics, activity participation, inflow time, housing situation, and other indicators [24, 38, 39]. The present study measured SSI in two dimensions from a factor analysis of eight questions and found that a stronger *will to integrate* had a significant positive impact on mobility during pregnancy and delivery in receiving areas, while *level of participation* did not show a significant impact. Since 2000, the Chinese government has been trying to promote social integration and decrease social exclusion for migrants by providing them more social welfare, insurance, and a generally migrant-friendly environment in the receiving areas. With resulting social integration, we expect an increase in pregnant migrants staying in receiving areas for most of their pregnancy period and giving birth there.

The World Health Organization believes it is necessary to make public health service delivery more migrant-friendly [40], and the right to health care for internal migrants has been raised in many developing countries [41, 42]. In 2012, the number of domestic migrants in China was approximately 236 million [25]. Pregnant migrant women’s health, which relates to the health of two generations, should thus be a focus of attention; however, there is still a gap between health care provision for pregnant migrants and those for local household–registered populations, as well as in health care utilization between them [43, 44]. Given this background, the results of this study should remind maternal health service providers to pay more attention to the increasing number of pregnant migrant women spending most of their pregnancy and/or giving birth in receiving areas. Maternal health resources should be allocated with consideration to the size and shifting trends in this population, especially for those who are inclined to stay in receiving areas during most or all of their pregnancy and those who choose receiving areas as delivery locations (who are in general also those who have their own housing, those migrating on only a small scale, those with longer migration time, and those with a strong will to integrate), since more attention has been paid to the size and demands of the household registered population to date [43, 44] . For younger women without their *own housing*, with a cross-province or cross-city *migration range*, and who have high mobility during pregnancy (MR and MS), the integrity and continuity of medical records between sending and receiving areas need to be improved (remembering that medical record systems are a general challenge for the efficiency of health care in many developing countries [45, 46]).

This study has some limitations that should be reflected here. First, the power of cause–effect relationships in a cross-sectional study is low. Our study could not securely demonstrate causal effects but does give clues as to the characteristics of migrant women with high mobility during pregnancy. Second, the study may have some selection bias. The sample is from a migrant population in 2012, based on which we reviewed the trends of the migrants’ mobility during the pregnancy and delivery locations of their first child between 1985 and 2011. Families that migrated in the early years but returned to their registered household in recent years were not included in the sampling frame. The lack of these families’ information may cause bias.

This study analyzed mobility patterns during pregnancy as well as selection of delivery locations among migrants in China using cross-sectional data for a large population. To the best of our knowledge, this is the first study to describe the changing patterns of mobility during pregnancy based on representative large-scale national population data from China. Moreover, the study focused on internally migrating pregnant women who may face higher risks of adverse pregnancy outcomes due to less health care utilization in the perinatal period. As we know, without prenatal examinations, a pregnant woman’s conditions such as hypertension, hyperglycemia, and some infectious diseases cannot be diagnosed, not to mention treated in time, and fetus position and health status cannot be assessed. The present study described the scale, characteristics, and change trends of the population in China, which can provide a reference for maternal health service providers in developing countries.

## Conclusions

Our research indicated that the percentage of migrants always or mainly staying in receiving areas during pregnancy rose steadily between 1985 and 2011 in China. The percentage of migrants who were mobile during pregnancy increased and then decreased, while the percentage of respondents who chose to deliver in receiving areas increased. Individual and family characteristics, including age, owning housing, rural/urban sending areas, migration range, migration time, and will to integrate were extensively associated with mobility patterns and delivery location choice. Maternal health care providers might consider the size and change trends of this population.

## Additional file

Additional file 1: Introduction of sampling methods of “Monitoring Data of Chinese Migrants”. (DOCX 21 kb)

## Abbreviations

AR: Always staying in the receiving area
AS: Always staying in the sending area
EC: Engel coefficient
MR: Mainly staying in the receiving area
MS: Mainly staying in the sending area
RA: Receiving area
SA: Sending area
SES: Socio-economic status
SSI: Status of social integration
