# Supplementary material for: Mobility patterns and associated factors among pregnant internal migrant women in China: a cross-sectional study from a National Monitoring Survey
Source: BMC Pregnancy Childbirth. 2018 May 15;18:165. doi: 10.1186/s12884-018-1813-2 (PMC5952471; doi:10.1186/s12884-018-1813-2)
Supplement: Supplementary file 1 — Introduction of sampling methods of “Monitoring Data of Chinese Migrants”. (DOCX 21 kb) [file 12884_2018_1813_MOESM1_ESM.docx]

Additional file 1:

Introduction of sampling methods of “Monitoring Data of Chinese Migrants”.

The 2012 Migrant Dynamics Monitoring Survey in China adopted three-stage (town/subdistrict, village/community, and individual) sampling with probability proportional to size (PPS) sampling methods.

1、Sample size estimation and distribution


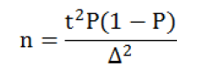


The sample size equation estimated the value of △ for a simple random sample as 5%-10% and that of t as 2. For the size of a complexly designed sample, the design effect was 2–3. From the main indicators of the 2011 Migrant Dynamics Monitoring Survey (e.g., ratio of the unmarried, employment ratio, rate of pension insurance participation), the basic sample size required for each stratum, with province as the unit, was estimated to be 4,000. However, each province increased the sample size according to the representativeness required, treating 4000 observations as the basic requirement. For example, 11,000 people were added for Shanghai, classified as a city in the 2012 Study on Migrants in Megacities. Eventually, various provinces as of 2012 were classified into six groups with sample sizes of 15,000, 12,000, 10,000, 8,000, 6,000, and 4,000 people, respectively. The total sample size for the whole country was expected to be 159,000.

2. Stratification and Weighting

The country was stratified into 31 provinces across China, with disproportionate sampling used for each provincial unit (as illustrated above). Therefore, the sampling ratios of each provincial unit needed to be weighted for the calculation of national indicators. Units at provincial level adopted PPS sampling, so sample weighting was not needed. During sampling, the sample size was determined according to the probability proportional distribution, which was based on data from the 2012 Statistics Report on National Migrant Populations.

3. PPS sampling

The first phase: Towns/subdistricts were selected in each province. (1) Estimating the size in each PPS group in the town/subdistrict stratum. Two hundred people were confirmed in each group according to factors influencing group size, such as sampling size in the second phase and the proportion of 15-59 aged population in total migrants. (2) Calculating the number of migrant groups in each town/subdistrict, that is, the number of migrants in each town/subdistrict divided by 200. (3) Sorting and coding each town/subdistrict. (4) Determining the actual number of people to be surveyed at each minimum unit (village/community). Considering that each investigator could survey about 20 people every 3–5 working days in each survey unit, we set the number of people to be surveyed in each basic survey unit as 20. (5) Number of survey groups needed = estimated sample size /20. (6) The random number method was used to select the groups and the corresponding town/subdistrict. Towns/subdistricts with more people might be selected more than once.

The second phase: Among all selected towns/subdistricts, villages/communities were chosen. (1) Estimating the size in each PPS group in the village/community stratum. It was roughly estimated that the group size should be 150 people to guarantee the required minimum unit size (20 people aged 15-59). (2) Confirming the number of groups included for each village/community, that is, the number of migrants per village/community divided by 150. (3) Sorting and coding each village/community. (4) A certain number of groups were randomly selected from each town/subdistrict. The number of groups selected was equal to the number of times a town/subdistrict was selected in the first phase.

The third phase: Confirming the surveyed subjects among the selected groups. (1) Within the selected groups, subjects were categorized by living status as follows: “living alone,” “living in a group,” and “living in work shed or other temporary residence” (It was considered that characteristics such as age, gender, and occupation of migrants are closely related to living status. Most people living in collective dormitories were unmarried young people, and most of those living in temporary work sheds at locations such as construction sites were middle-aged men working in the construction sector. Those living sparsely in the community often belonged to a different age range than the former two groups. Thus, grouping the sample by living status helped operationalization and enhanced sampling efficiency). (2) A list of 100 qualified subjects (aged 15-59) was compiled according to the distribution of subjects across the living status subgroups. (3) After arranging the 100 subjects in order based on gender, age, and duration of staying, 20 people were sampled by systematic sampling. (4) Only one migrant aged between 15 and 59 was surveyed in each family.
